# Bioinformatics analysis of the transcriptional expression of minichromosome maintenance proteins as potential indicators of survival in patients with cervical cancer

**DOI:** 10.1186/s12885-021-08674-y

**Published:** 2021-08-18

**Authors:** Baojie Wu, Shuyi Xi

**Affiliations:** Shanghai Zerun Biotechnology Co., Ltd., Pilot Department, Building 9, 1690 Zhangheng Road Pudong, Shanghai, 201203 China

**Keywords:** Minichromosome maintenance proteins, Transcriptional expression, Cervical intraepithelial neoplasia, Cervical cancer, Functional enrichment, Survival and prognosis

## Abstract

**Background:**

As major regulators of DNA replication in eukaryotes, minichromosome maintenance (MCM) proteins play an important role in the initiation and extension of DNA replication. MCMs and their related genes may be new markers of cell proliferation activity, which is of great significance for the diagnosis and prognosis of cervical cancer.

**Methods:**

To explore the role of MCMs and their related genes in cervical cancer, various bioinformatics methods were performed. First, the ONCOMINE and UALCAN databases were used to analyze the mRNA expression of different MCMs. The Human Protein Atlas database was used to analyze the protein expression of MCMs in normal and tumor tissues. The potential clinical value of MCMs was evaluated using the UALCAN, Kaplan-Meier plotter and cBioPortal databases. Then, the related genes and key coexpressed genes of MCMs were screened using GEPIA2 and cBioPortal analysis. For these genes, we used Metascape and the DAVID database to perform Gene Ontology (GO) and Kyoto Encyclopedia of Genes and Genomes (KEGG) pathway enrichment analyses, construct the related molecular interaction network, and obtain the key subnetworks and related hub genes. The Kaplan-Meier plotter database was used for survival analysis of cervical cancer patients to evaluate and predict the potential clinical value of the hub genes. Moreover, multiple gene comparisons of the expression of MCMs and related genes in different cancer types also showed the clinical significance of these potential targets.

**Results:**

The mRNA and protein expression of MCMs increased in tumor tissue. Overexpression of MCM2/3/4/5/6/7/8/10 was found to be significantly associated with clinical cancer stage. Higher mRNA expression levels of MCM3/5/6/7/8 were found to be significantly associated with longer overall survival, and higher mRNA expression of MCM2/3/4/5/6/7/8 was associated with favorable OS. In addition, a high mutation rate of MCMs (71%) was observed. *MCM2*, *MCM4*, *MCM8*, *MCM3* and *MCM7* were the five genes with the most genetic alterations. In addition, the coexpressed genes and related genes of MCMs were successfully screened for enrichment analysis. These genes were significantly enriched in important pathways, such as the DNA replication, cell cycle, mismatch repair, spliceosome, and Fanconi anemia pathways. A protein-protein interaction network was successfully constructed, and a total of 13 hub genes (*CDC45*, *ORC1*, *RPA1*, *CDT1*, *TARDBP*, *RBMX*, *SRSF3*, *SRSF1*, *RFC5*, *RFC2*, *MSH6*, *DTL*, and *MSH2*) from 4 key subnetworks were obtained. These genes and *MCM2/3/4/5/6/7/8* might have potential clinical value for the survival and prognosis of cervical cancer patients.

**Conclusions:**

These findings promoted the understanding of the MCM protein family and clinically related molecular targets for cervical epithelial neoplasia and cervical cancer. Our results were helpful to evaluate the potential clinical value of MCMs and related genes in patients with cervical cancer.

## Background

The minichromosome maintenance (MCM) protein family is a group of proteins closely related to DNA replication and genome stability [1]. Highly conserved MCM complex proteins may have helicase activity and are essential for the initiation of DNA replication. MCM complex proteins contain ATPase domains, and energy is harnessed to affect DNA unwinding [2]. There are ten characterized homologous MCM genes. MCM2–7 form a replicative helicase complex [3], and MCM8 and MCM9 form a dimer involved in homologous recombination repair [4]. The ninth gene that encodes an MCM domain is named MCM domain-containing 2 (MCMDC2) [5]. MCM10 is a dynamic scaffold at eukaryotic replication forks [6].

**Fig. 1:**
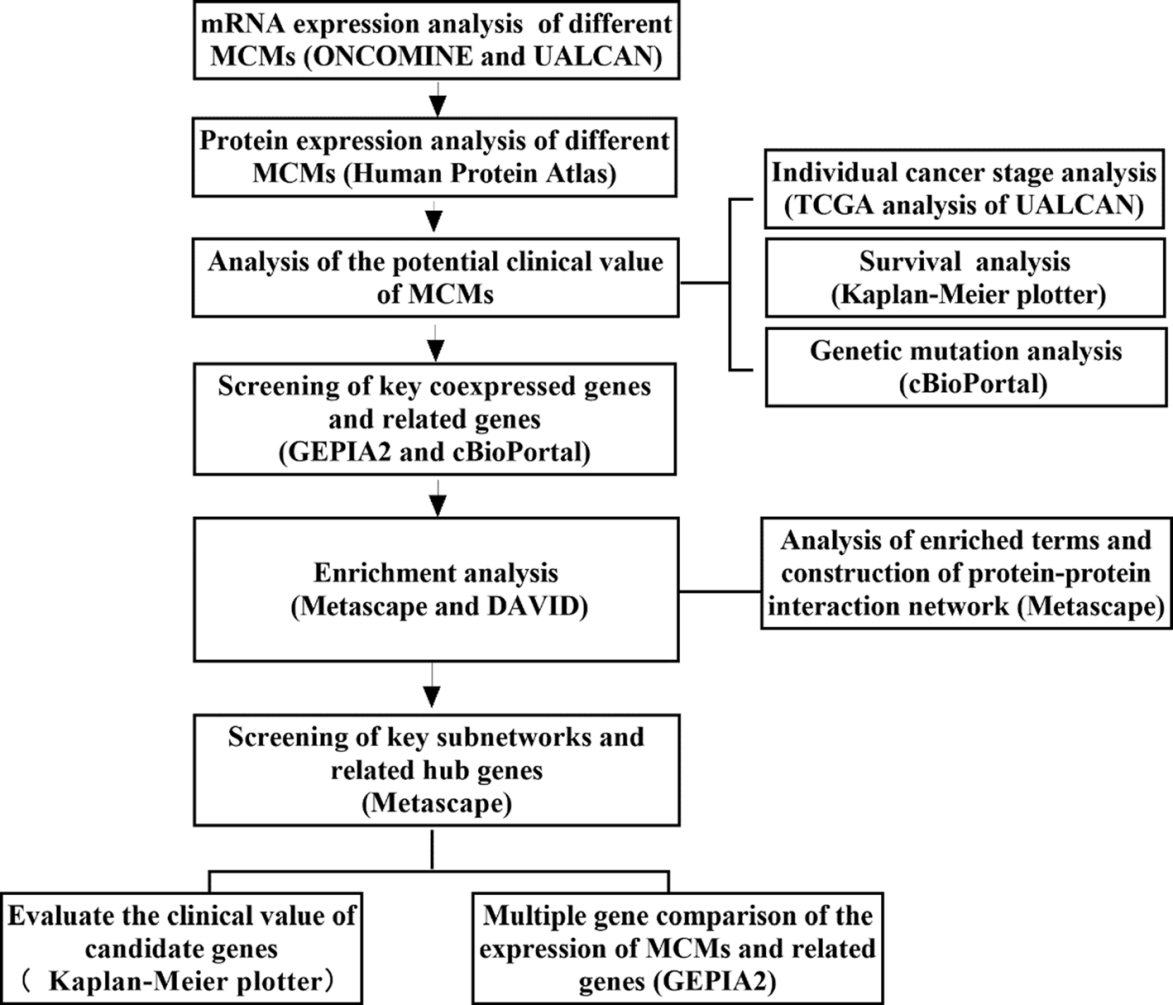
Flowchart of the integrated analysis

It has been reported that the expression of MCMs in the cell proliferation cycle is one of the important factors of DNA replication initiation and extension, and their positive expression is an important marker of cell proliferation [7]. Their expression levels are related to the proliferation and differentiation of tumor cells and can accurately reflect the proliferation activity of cells. MCMs have great reference value in the early diagnosis, classification and prognosis of clinical tumors [8, 9]. Therefore, it is necessary to strengthen the research on the basic theory and clinical application value of MCMs, including their mechanism of protein action, their expression characteristics and their related genes in cervical cancer tissue, as well as their value in clinical diagnosis and differential diagnosis. At present, there are few related studies in these areas. In this study, the roles of MCMs and related genes in cervical cancer were investigated by a variety of bioinformatics methods. By analyzing the mRNA and protein expression of MCM family members, their potential clinical value in cervical cancer was analyzed. The workflow of this study is shown in Fig. 1.

## Methods

### Expression of different MCMs in cervical cancer patients

To explore the distinct prognostic and potential therapeutic values of different MCM members in cervical cancer patients, the mRNA expression of different MCMs was analyzed by the ONCOMINE database [10] (www.oncomine. org) and UALCAN [11] (http://ualcan.path.uab.edu). After analyzing the mRNA expression, the protein expression of different MCMs in cervical cancer was explored by the Human Protein Atlas [12–14] (HPA, http://www.proteinatlas.org) database, and the results of immunohistochemistry from HPA showed the expression of MCMs in normal tissues and tumor tissues. The data used for analysis were from databases, and the expression results of different MCMs can provide a reference for evaluating their potential clinical value.

### Potential clinical value of MCMs

After mRNA and protein expression analyses, the relationship between the mRNA expression of MCMs and the clinicopathological parameters of patients, such as individual cancer stages, was assessed by performing The Cancer Genome Atlas (TCGA) database analysis via UALCAN. The data used for analysis were from the database, and the analysis results showed the potential value of different MCMs in clinical pathology. Moreover, the survival of patients was analyzed, and specific MCMs related to better prognosis were identified.

Furthermore, the Kaplan-Meier plotter database [15, 16] (http://kmplot.com/analysis/) was used to analyze the prognostic value of the mRNA expression of different MCMs in cancer patients. The correlation between the mRNA expression of MCMs and the prognosis of patients with cervical cancer was explored and analysed. MCMs useful for predicting the survival of patients with cervical cancer were identified.

After mRNA expression of specific MCMs was found to be significantly associated with patient prognosis, genetic alterations in MCMs and their associations with overall survival (OS) and disease-free survival (DFS) of cervical squamous cell carcinoma (CESC) patients were analyzed by the cBioPortal database [17, 18] (www.cbioportal.org). Analysis of genetic alterations promoted the exploration and understanding of different MCMs in CESC, identified MCMs that are prone to alteration and provided information support for genetic alterations of MCMs in cervical cancer. In addition, key coexpressed genes of MCMs in CESC (TCGA, PanCancer Atlas) were screened and analyzed via a Venn diagram (http://bioinformatics.psb.ugent.be/webtools/Venn/).

### Similar gene detection and enrichment analysis of related genes in CESC tumors

After the analysis of the ONCOMINE, UALCAN, HPA, Kaplan-Meier plotter and cBioPortal databases, the functions of the MCMs with potential value and their related genes in CESC tumors were further enriched and explored. Genes that had a similar expression pattern to MCMs in CESC tumors were analyzed by GEPIA2 [19] (http://gepia2.cancer-pku.cn/#similar). Then, the key coexpressed genes and related genes of MCMs in CESC tumors were analyzed by Metascape [20] (https://metascape.org/gp/index.html). The pathways and process enrichment of these genes were determined [21]. Database for Annotation, Visualization and Integrated Discovery (DAVID, https://david.ncifcrf.gov/) was also used to verify the biological processes, cellular components, molecular functions and KEGG pathways of these genes [22–24].

### Protein-protein interaction (PPI) network construction and screening of hub genes

Next, the network of enriched terms and the PPI network were also analyzed by Metascape [25–27]. Then, key subnetworks and related hub genes were obtained, and the Kaplan-Meier plotter database was used for survival analysis of cervical cancer patients to evaluate and predict the potential clinical value of the hub genes [28]. Moreover, multiple gene comparisons of the expression of MCMs and related genes in different cancer types were performed to show the clinical significance of these potential targets by GEPIA2. In particular, MCMs and their related genes that are involved in the progression of cervical cancer might provide potential targets for the clinical prevention, treatment, and effective prognostication of cervical cancer.

## Results

### Expression of different MCMs in cervical cancer patients

**Fig. 2:**
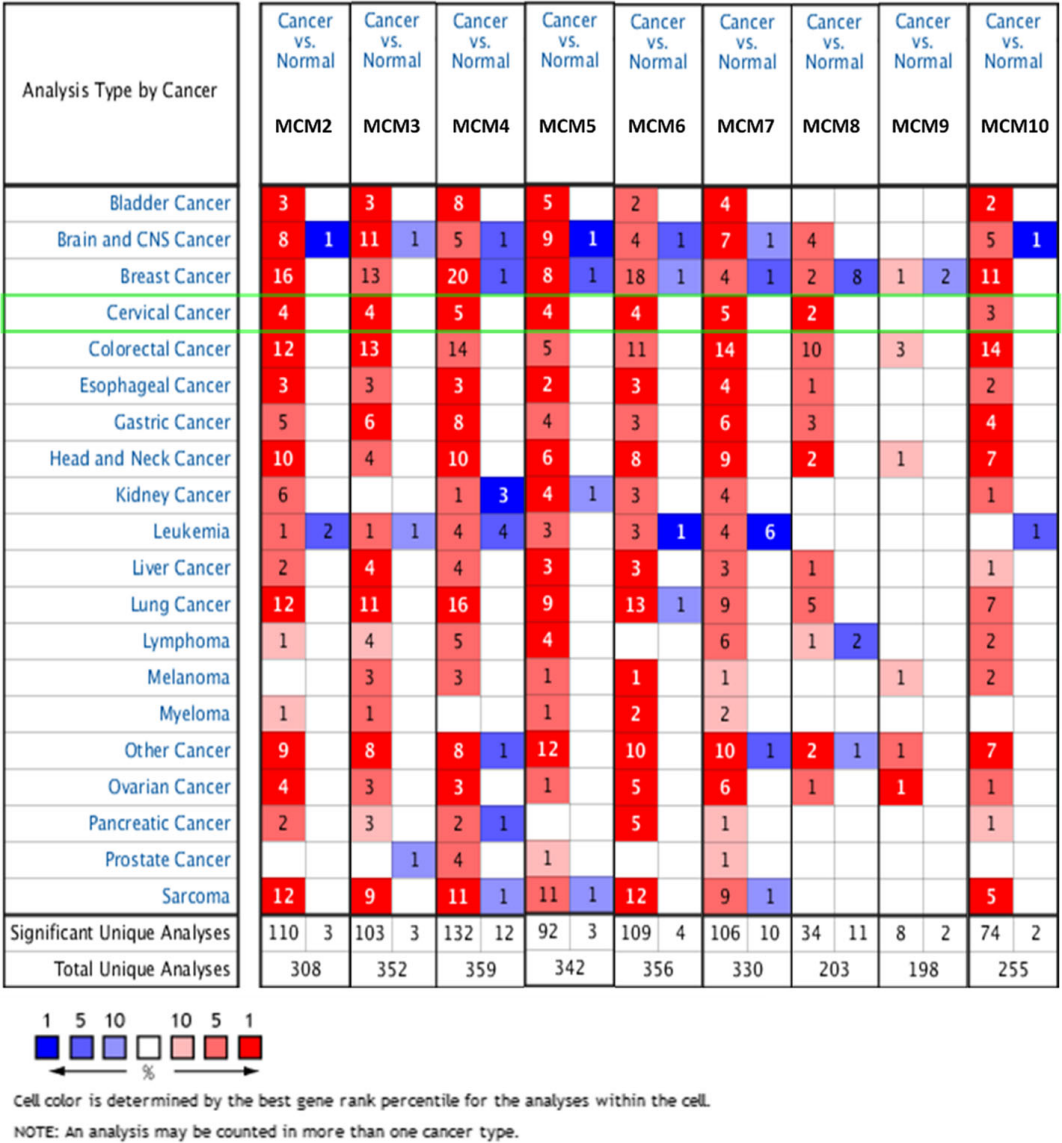
Transcriptional expression of MCMs in different types of cancer (ONCOMINE). Differences in transcriptional expression were compared by t-tests. The cutoff *p*-value, fold change, and other settings were as follows: *p*-value: 0.01, fold change: 1.5, gene rank: top 10%, data type: mRNA

**Fig. 3:**
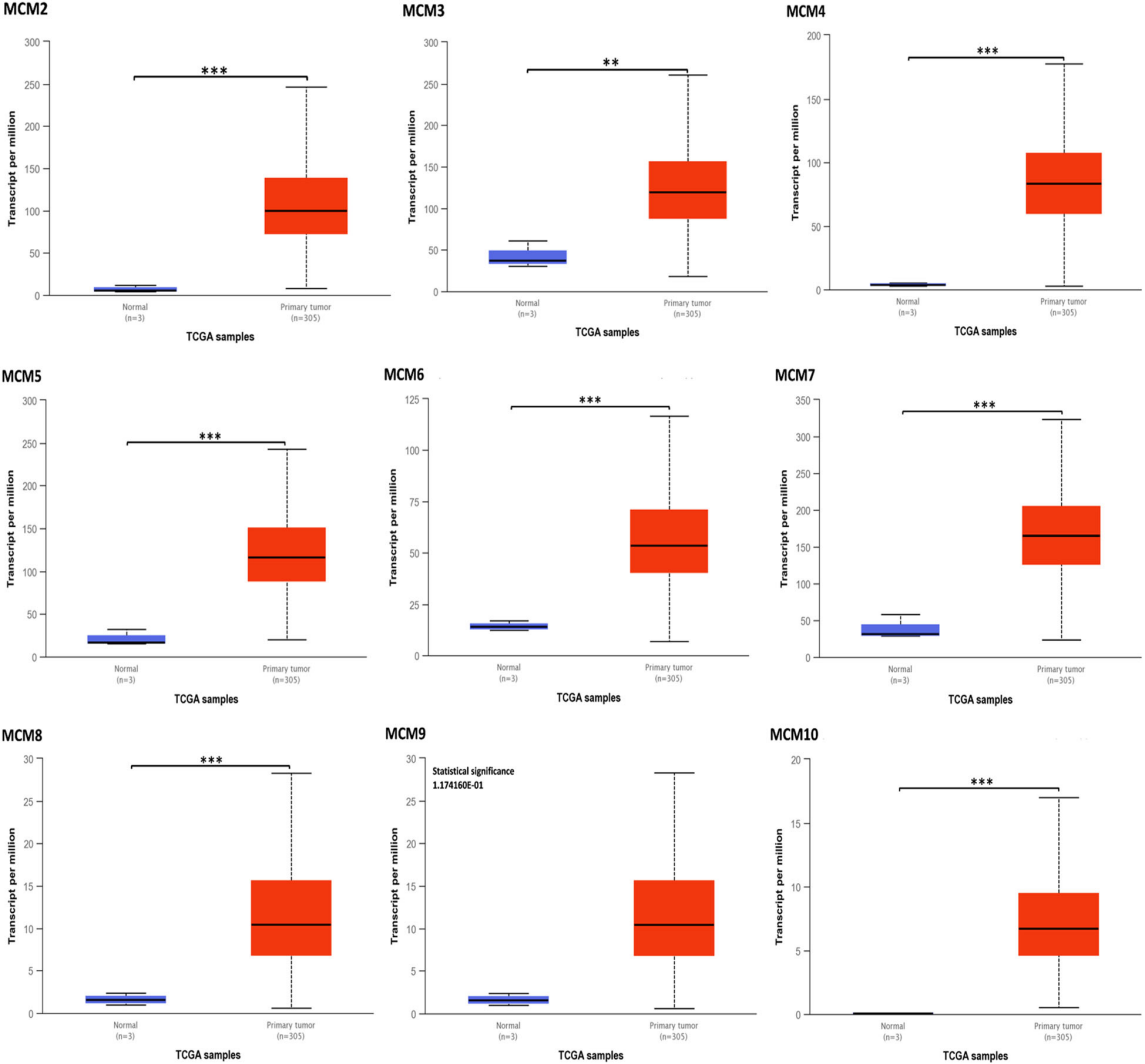
mRNA expression of MCMs in CESC tissues and normal tissues (UALCAN). *p < 0.05, **p < 0.01, ***p < 0.001

**Fig. 4:**
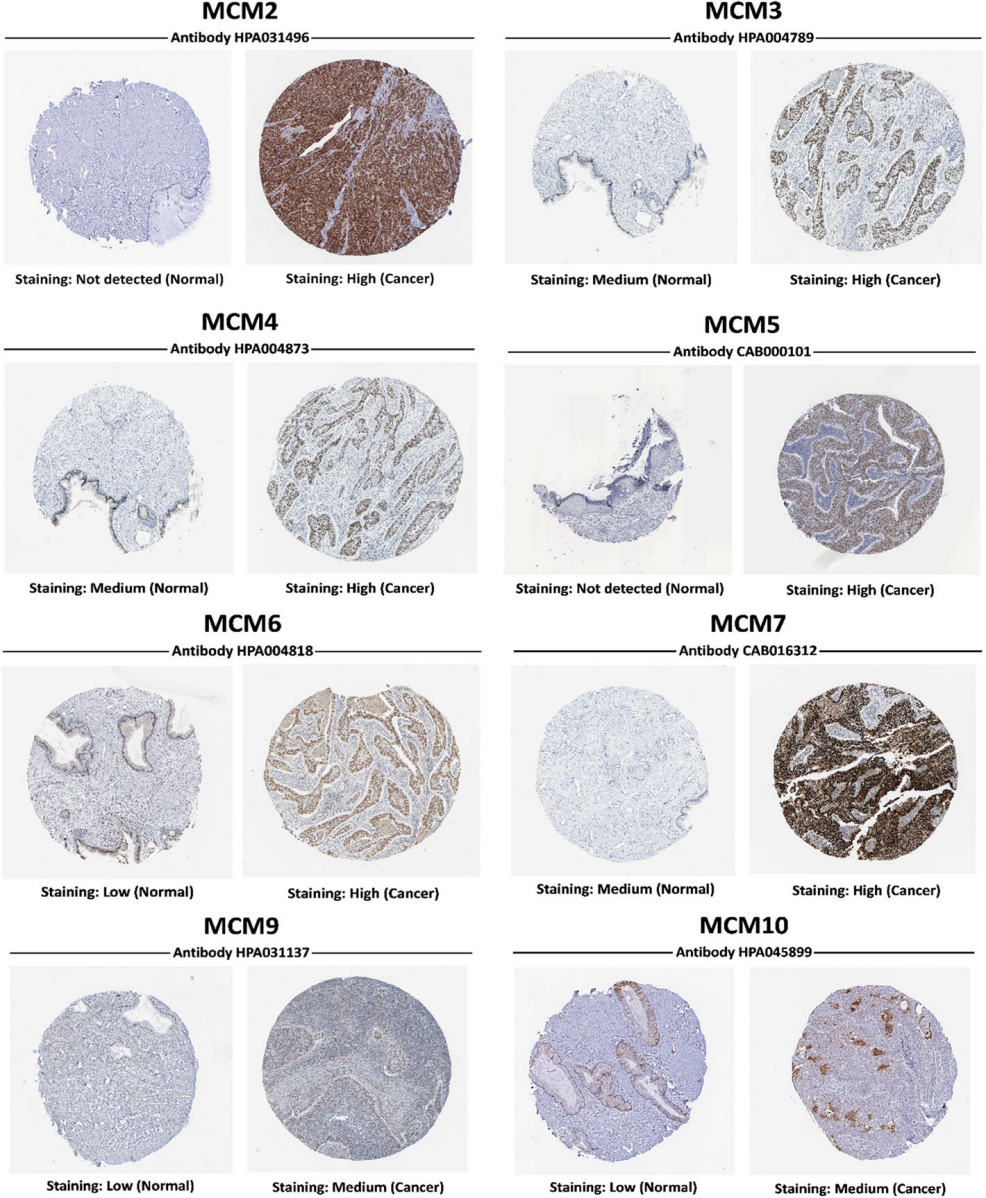
Differential expression of MCMs in cervical cancer tissues and normal tissues (Human Protein Atlas). MCM8: No data in HPA database

**Fig. 5:**
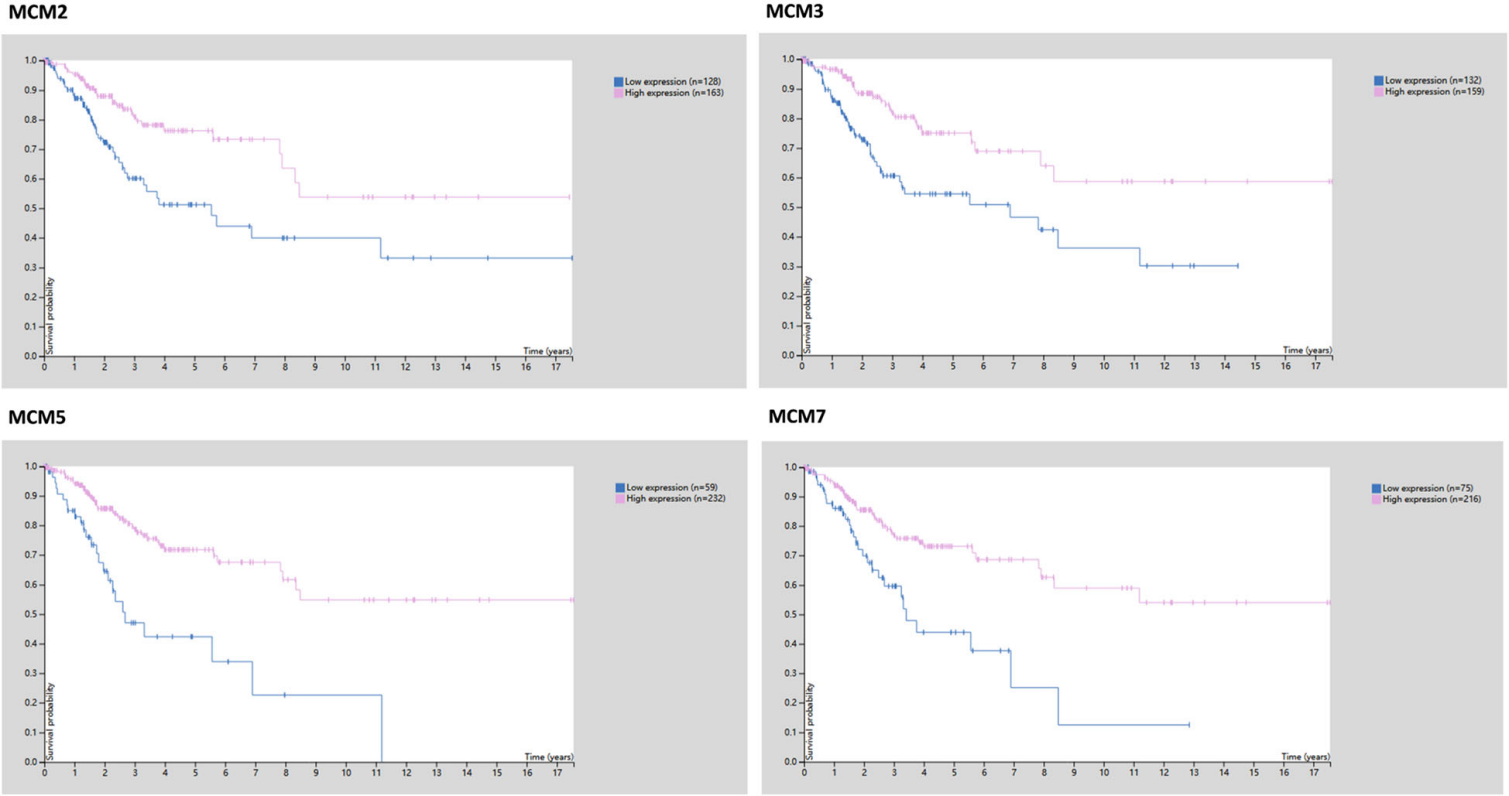
Prognostic markers of cervical cancer (favorable) in the Human Protein Atlas database (*p* < 0.001)

As shown in Fig. 2 and Table 1, the mRNA expression levels of MCM2/3/4/5/6/7/8/9/10 in cervical cancer tissues and normal tissues were compared using the ONCOMINE database [29–32], and MCM2/3/4/5/6/7/8/10 expression was significantly increased in tumor tissues. Then, the mRNA expression of MCM2/3/4/5/6/7/8/9/10 was further analyzed by the UALCAN database. As shown in Fig. 3, the mRNA expression of MCM2/3/4/5/6/7/8/10 in tumor tissues was significantly higher than that in normal tissues (*p* < 0.05), while the expression of MCM8/9/10 in tumor tissues was lower than that of other MCMs (MCM2/3/4/5/6/7). However, the expression of MCM9 in tumor tissues was not significant. In addition to the above analysis, immunohistochemical information was obtained from the HPA database to analyze the protein expression of MCMs in normal and tumor tissues (Fig. 4). MCM2 and MCM5 were not detected in normal tissues, but their high expression was observed in tumor tissues. The expression of MCM6/9/10 was low in normal tissues, but medium and high protein expression was observed in tumor tissues. In addition, medium protein expression of MCM3/4/7 was observed in normal tissues, and high protein expression was observed in tumor tissues. Moreover, high expression of MCM2/3/5/7 was associated with good survival and prognosis (Fig. 5).

**Table 1:** Significant changes in MCM expression at the transcription level between cervical cancer tumor and normal tissues (ONCOMINE)

MCMs	Types	Fold Change	*P*-value	T-test	References
MCM2	CESC	5.984	2.3E-17	12.158	Scotto Cervix 2 [29]
	CESCE	5.714	3.12E-13	13.368	Zhai Cervix [30]
	CC	5.219	3.28E-14	11.233	Pyeon Multicancer [31]
	CESC	2.76	1.00E-05	11.995	Biewenga Cervix [32]
MCM3	CC	4.18	2.84E-13	11.188	Pyeon Multicancer [31]
	CESC	2.613	1.05E-10	7.947	Scotto Cervix 2 [29]
	CESCE	1.73	2.44E-07	7.464	Zhai Cervix [30]
	CESC	2.589	1.48E-06	12.088	Biewenga Cervix [32]
MCM4	CESCE	2.100	3.03E-11	10.279	Zhai Cervix [30]
	HG-CIN	2.161	0.001	3.672	Zhai Cervix [30]
	CC	4.103	1.48E-13	11.000	Pyeon Multicancer [31]
	CESC	3.175	3.90E-10	7.686	Scotto Cervix 2 [29]
	CESC	2.806	6.06E-06	12.289	Biewenga Cervix [32]
MCM5	CESC	4.399	3.80E-14	10.142	Scotto Cervix 2 [29]
	CC	3.623	3.49E-11	9.255	Pyeon Multicancer [31]
	CESCE	9.905	1.59E-05	7.196	Zhai Cervix [30]
	CESC	2.550	7.54E-06	13.686	Biewenga Cervix [32]
MCM6	CESC	3.731	6.25E-13	9.510	Scotto Cervix 2 [29]
	CC	4.004	3.28E-12	10.400	Pyeon Multicancer [31]
	CESCE	2.655	2.93E-07	8.894	Zhai Cervix [30]
	CESC	3.130	1.03E-07	17.000	Biewenga Cervix [32]
MCM7	CESC	3.728	5.77E-11	8.050	Scotto Cervix 2 [29]
	CESCE	3.233	5.10E-07	8.604	Zhai Cervix [30]
	HG-CIN	2.480	0.003	3.577	Zhai Cervix [30]
	CC	2.768	2.90E-09	7.790	Pyeon Multicancer [31]
	CESC	2.666	1.54E-07	13.539	Biewenga Cervix [32]
MCM8	CC	4.454	3.82E-14	11.480	Pyeon Multicancer [31]
	CESC	1.907	0.00000942	11.006	Biewenga Cervix [32]
MCM9					Need further study
MCM10	CESCE	2.287	0.00000367	5.779	Zhai Cervix [30]
	CESC	4.046	1.47E-06	5.463	Scotto Cervix 2 [29]
	CC	2.176	2.74E-08	7.154	Pyeon Multicancer [31]

Cervical squamous cell carcinoma epithelia: CESCE; High-grade cervical squamous intraepithelial neoplasia epithelia: HG-CIN; Cervical cancer: CC; Cervical squamous cell carcinoma: CESC

### Potential clinical value of MCMs and screening of coexpressed genes

**Fig. 6:**
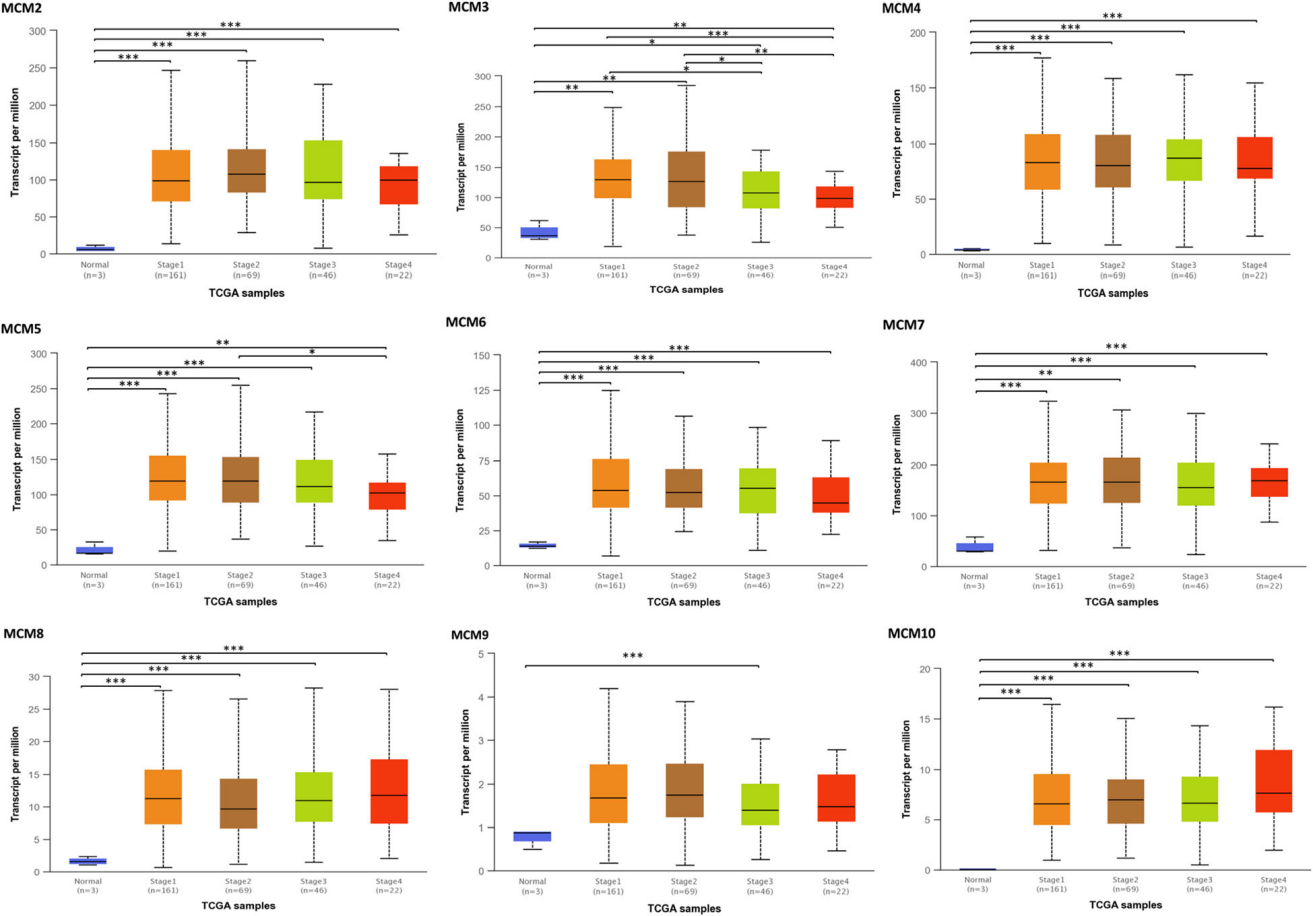
Relationship between mRNA expression of MCMs and individual cancer stages in CESC, *p < 0.05, **p < 0.01, ***p < 0.001 (UALCAN)

**Fig. 7:**
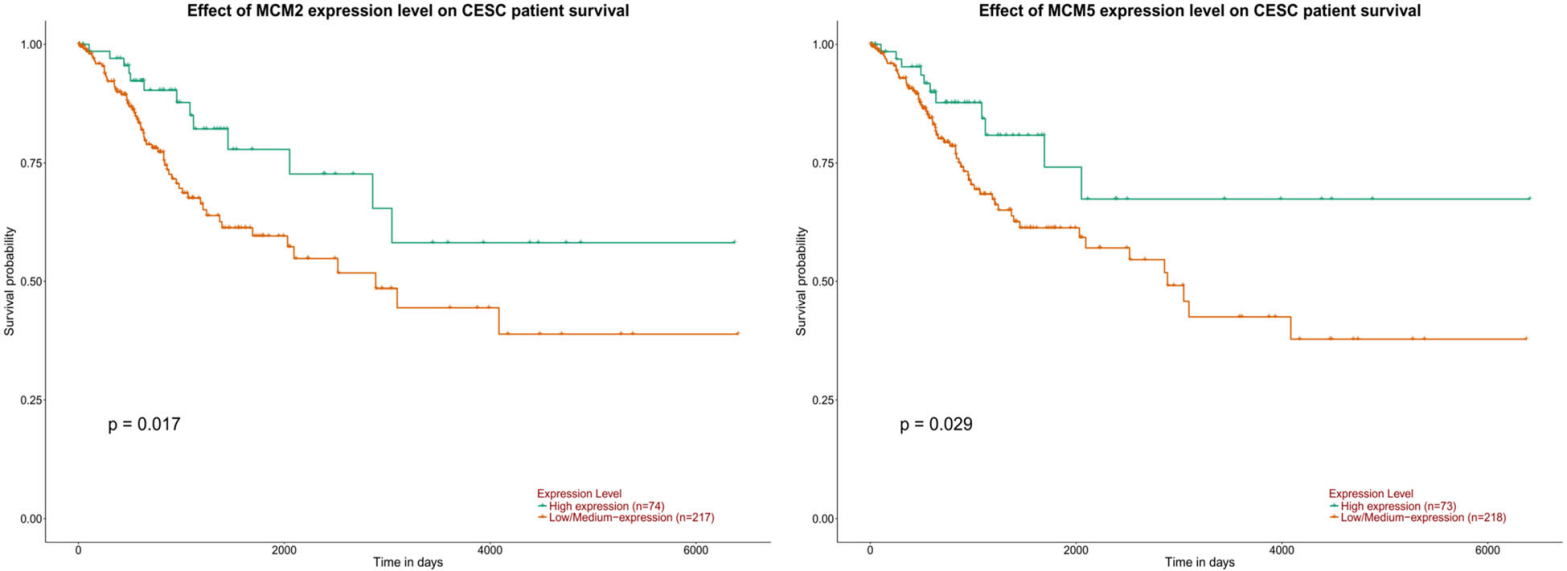
Effect of MCM2 and MCM5 expression levels on CESC patient survival (UALCAN)

As shown in Fig. 6, the results showed that the mRNA expression of MCMs was significantly correlated with individual cancer stages in the UALCAN database, and cancer patients (stage 1-stage 4) had higher mRNA expression of MCMs than normal controls. The effects of MCM2 and MCM5 expression are shown in Fig. 7. High expression of MCM2 and MCM5 had a significant impact on patients, and they might be better targets to promote the good survival and prognosis of patients.

**Fig. 8:**
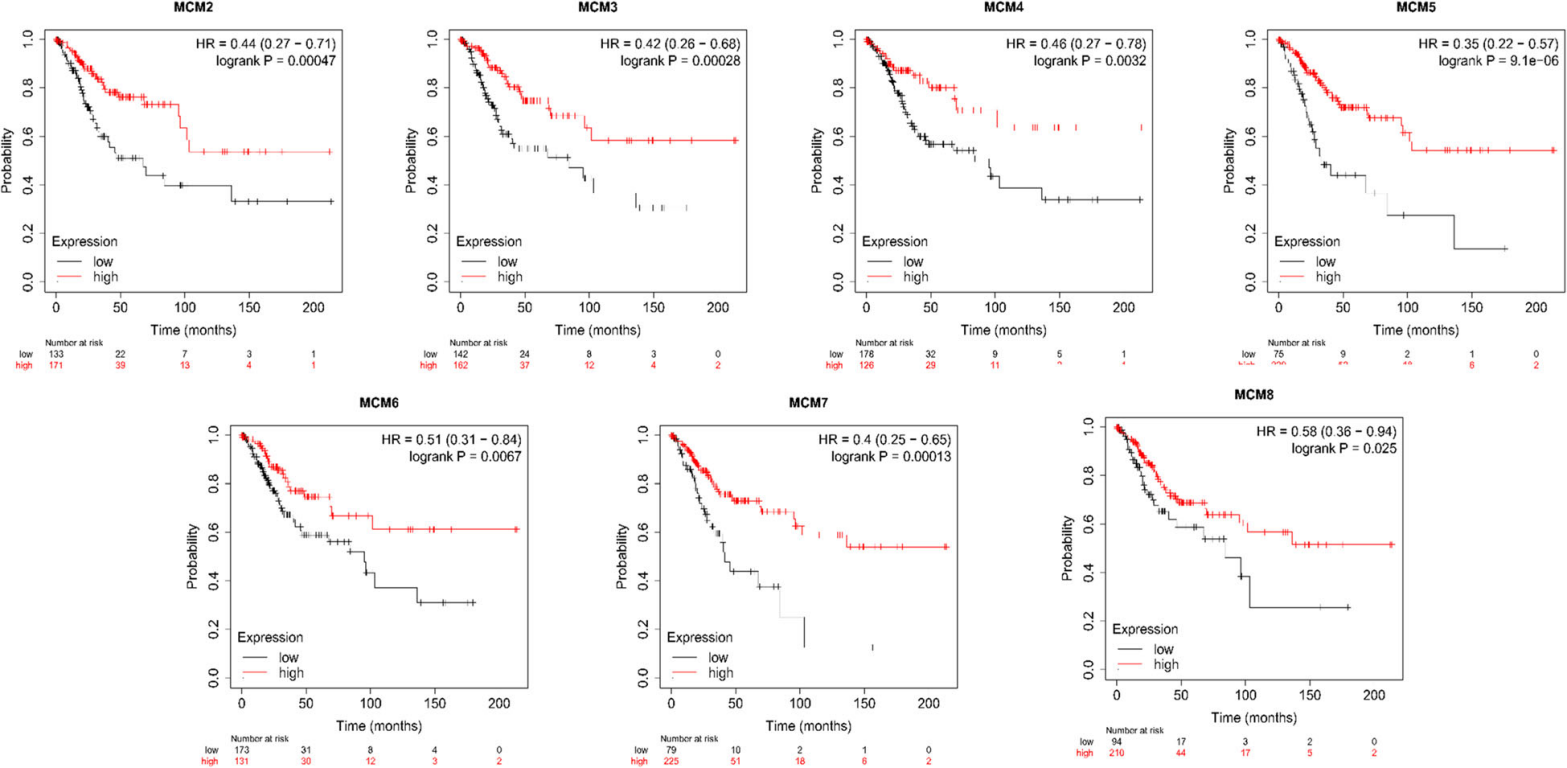
Prognostic value of MCM2/3/4/5/6/7/8 mRNA expression in CESC patients (Kaplan-Meier plotter database)

**Fig. 9:**
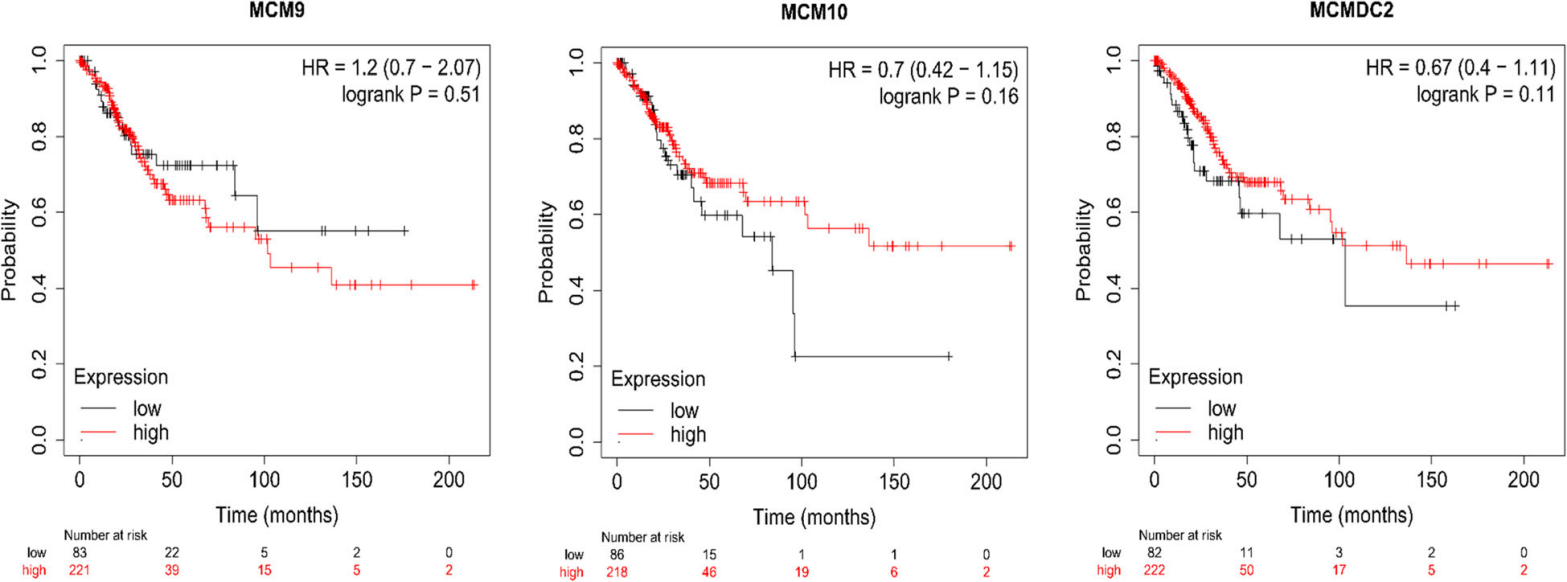
Prognostic value of the mRNA expression of MCM9/10 and MCMDC2 in CESC patients (Kaplan-Meier plotter database)

Furthermore, the prognostic value of the mRNA expression of MCMs was evaluated using the Kaplan-Meier plotter database (Fig. 8). The mRNA expression of MCM2/3/4/5/6/7/8 was significantly associated with CESC patient prognosis, and the results showed that higher mRNA expression of MCM2/3/4/5/6/7/8 was associated with favorable OS in CESC patients. Moreover, higher mRNA expression of MCM3/5/6/7/8 was significantly associated with longer OS, but the mRNA expression of MCM9, MCM10 and MCMDC2 had no significant effect on the prognosis of CESC patients (Fig. 9). These results indicated that MCM2/3/4/5/6/7/8 may serve as useful biomarkers for CESC patients.

**Fig. 10:**
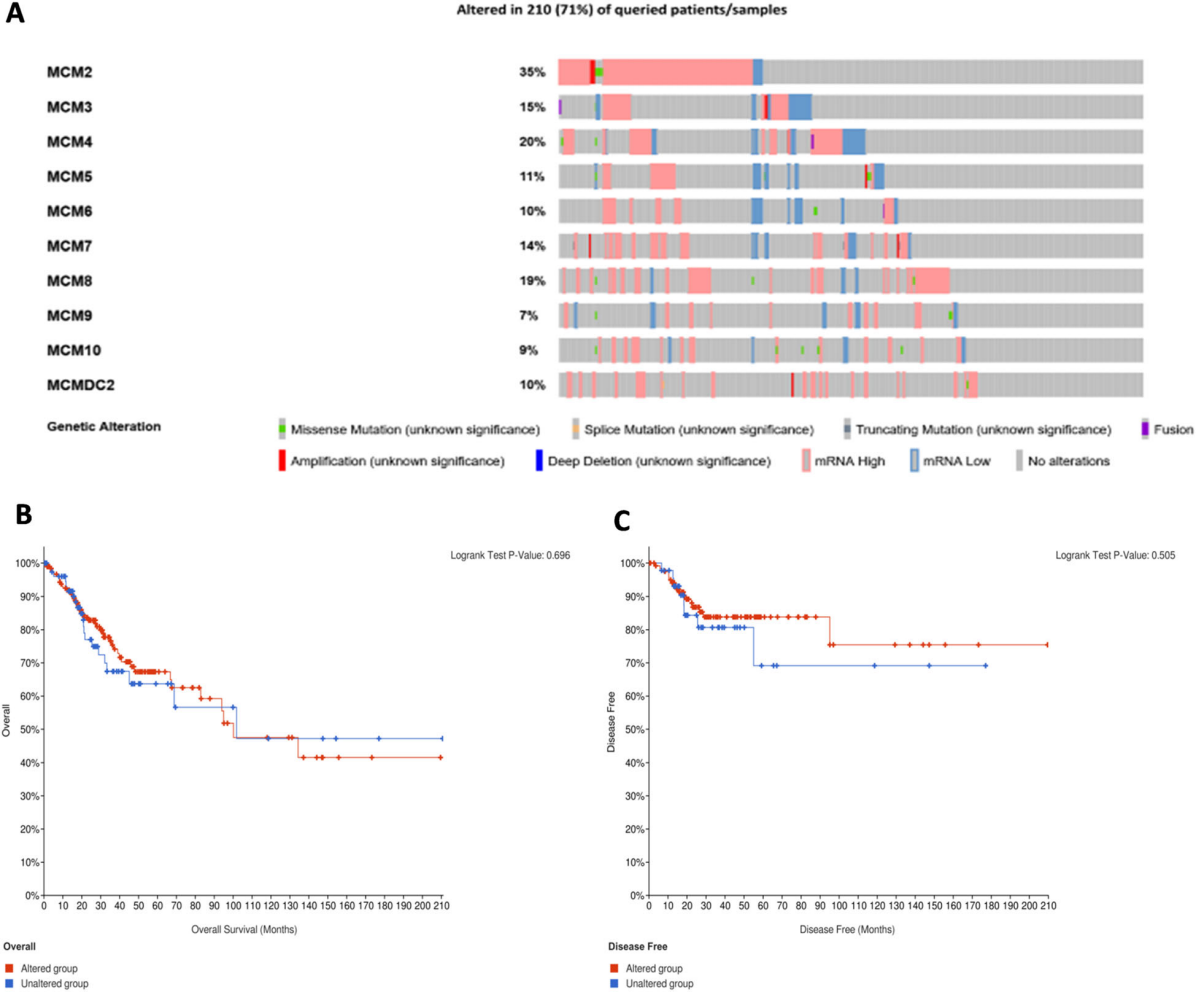
Analysis of MCMs in CESC patients (cBioPortal). **A**. Genetic mutations; **B**. Relationships between MCM gene mutations and OS and DFS in CESC patients (cBioPortal)

In particular, genetic mutations in MCMs and their associations with OS and DFS were explored by the cBioPortal database. The cBioPortal analysis showed a high mutation rate (71%) of MCMs in CESC patients (TCGA, PanCancer Atlas). *MCM2*, *MCM4*, *MCM8*, *MCM3* and *MCM7* were the top five genes with the highest genetic alterations, and the mutation rates were 35, 20, 19, 15 and 14%, respectively (Fig. 10). Moreover, the results showed that genetic alterations in MCMs were not significantly associated with longer OS or DFS in CESC patients.

Furthermore, to deeply explore the potential clinical value of MCMs, their coexpressed genes were analyzed using the cBioPortal database. The coexpressed genes of *MCM2/3/4/5/6/7/8* (top 25 correlated genes) were screened, and the results of intersection analysis are shown in Table 2. A total of 9 intersections and 16 key coexpressed genes (*MCM5* [33], *CHAF1B*, *MCM2*, *GINS2*, *MCM6*, *RFC5*, *FANCC*, *TIMELESS*, *CLSPN*, *BRIP1*, *RBL1*, *CDT1*, *RFC2*, *CDC45*, *ORC1*, and *TOP2A*) were obtained for further study.

**Table 2:** Intersection analysis of the coexpressed genes of MCMs in CESC (cBioPortal)

Groups	Total	Intersection elements
MCM2/6/7	1	*MCM5*
MCM3/5/6	1	*CHAF1B*
MCM5/6/7	2	*MCM2, GINS2*
MCM3/5	1	*MCM6*
MCM3/6	3	*RFC5, FANCC, TIMELESS*
MCM4/8	3	*CLSPN, BRIP1, RBL1*
MCM5/7	3	*CDT1, RFC2, CDC45*
MCM6/7	1	*ORC1*
MCM6/8	1	*TOP2A*
MCM2	24	*HMCES, COMMD2, GMPS, RYK, PIK3R4, MSL2, UMPS, DBR1, TBCCD1, HLTF, ZXDC, TOPBP1, TFDP2, RFC4, NAA50, EEFSEC, ACAD9, ARMC8, MBD4, RUVBL1, H1FX, SEC22A, ISY1, ASTE1*
MCM3	20	*PRSS16, DEK, ORC3, PPP2R5D, CHAF1A, CDC5L, TJAP1, CENPQ, CCHCR1, MAD2L1BP, MMUT, NUP85, NASP, CDKAL1, KIFC1, OARD1, GMNN, LOC730101, KLHDC3, PRIM2*
MCM4	22	*UBR5, CKAP2L, CCDC107, GTF2A1, MTBP, TAF2, RIF1, CCNE2, NCOA2, WDHD1, ATAD2, KNL1, PRR11, TGS1, SMC3, FKBP2, WASHC5, RAD21, VPS13B, PRKDC, DHX9, ARMC1*
MCM5	18	*TFIP11, FANCE, EWSR1, XRCC6, ESS2, EIF3D, NCAPH2, PICK1, SLC30A1, L3MBTL2, DMC1, POLA2, NOL12, CENPM, POLD1, RBX1, MCM7, TBC1D22A*
MCM6	16	*TYMS, CDCA7, MCM3, RIBC2, MTHFD1, DTL, UHRF1, PRIM1, PCNA, SMPD4, DNMT1, UNG, USP1, WDR76, MSH6, HAT1*
MCM7	18	*TRIP6, PIN1, KIF22, SRRT, LAMTOR4, DCTPP1, PDAP1, MDH2, NSUN5, ORC5, GNB2, RAD54L, MEPCE, MYBL2, CDCA5, CPSF4, BUD31, POP7*
MCM8	21	*ALMS1, CYB5RL, CSNK2A3, ESF1, XRN2, DHX35, ATAD5, RALGAPB, ANKEF1, NANP, GINS1, FAM217B, BARD1, SLX4IP, RPRD1B, PTPRA, RBBP9, TAF1, STK35, ATRN, ADNP*

### Detection and enrichment analysis of related genes

**Fig. 11:**
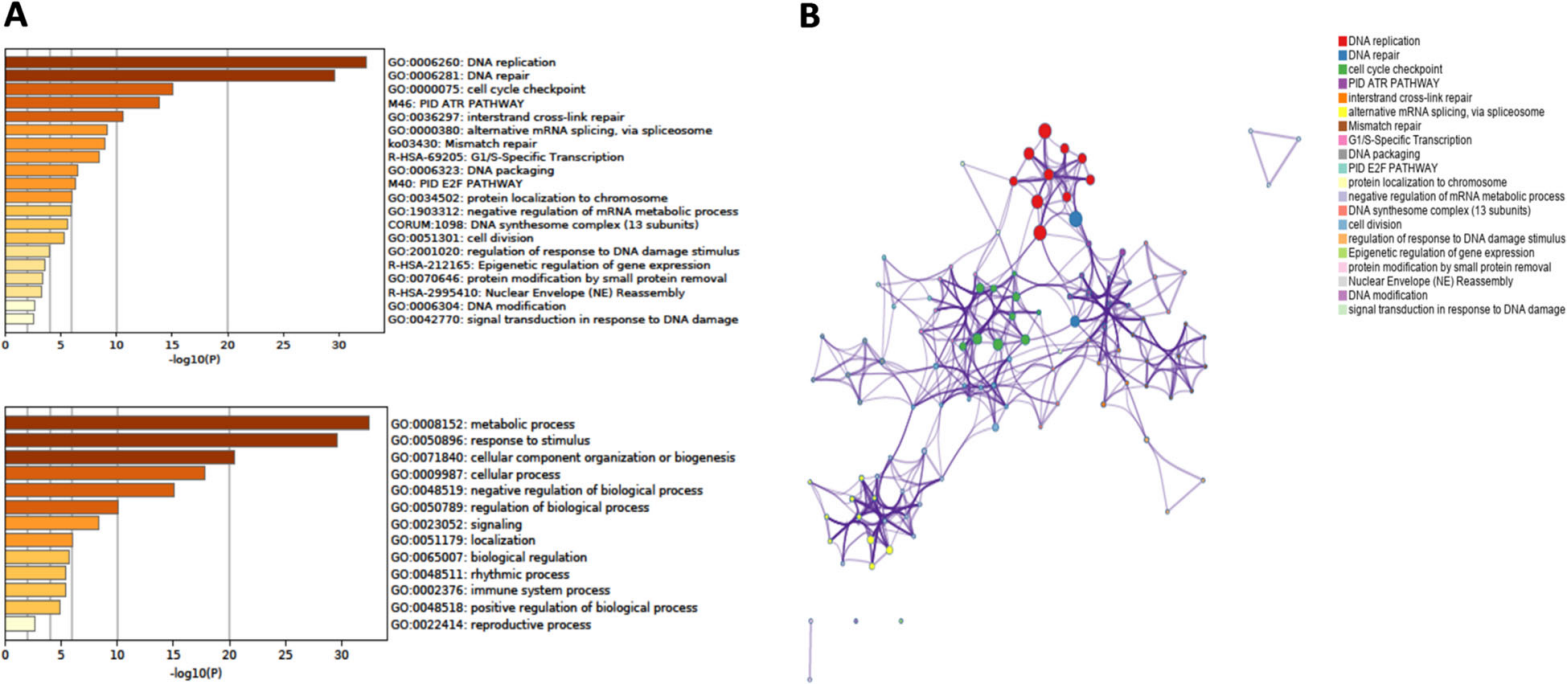
Enrichment results of the hub genes. **A**. Bar graph of the enriched terms (the top 20 clusters) and the top-ranked Gene Ontology biological processes; **B**. Network of the enriched terms colored by cluster ID. Nodes that share the same cluster ID are typically close to each other (Metascape)

Next, the top 50 genes related to MCMs were identified by GEPIA2. The 57 selected genes (as shown in Table 3) and *MCM7/8* were analyzed by Metascape. The pathway and process enrichment analysis results are shown in Fig. 11. The results showed that MCMs and related genes (59 total) were mainly enriched in DNA replication, DNA repair, the cell cycle, cell division and expression regulation. These genes were also analyzed by the DAVID database, and the Gene Ontology (GO) and Kyoto Encyclopedia of Genes and Genomes (KEGG) enrichment results (*p*-value< 0.05, false discovery rate < 0.05) are shown in Table 4, Table 5, Table 6 and Table 7. The MCM-related genes were significantly enriched in 21 biological processes, 5 cellular components, 14 molecular functions and 5 important KEGG pathways.

**Table 3:** Intersection analysis of the related genes and key coexpressed genes of MCMs in CESC

Groups	Total	Intersection elements
Common genes	9	*RFC5, MCM5, MCM6, TIMELESS, FANCC, MCM2, ORC1, CLSPN, BRIP1*
Key coexpressed genes	7	*CHAF1B, RBL1, GINS2, TOP2A, CDT1, RFC2, CDC45*
Related genes	41	*PPM1D, RBBP4, ZWINT, TMPO, TOPBP1, U2AF2, DEK, SRSF3, MCM3, HCFC1, MSH2, TUBA1B, DCAF11, THRAP3, SART3, CPSF6, MIS18BP1, CTCF, SAP130, LMNB1, ATAD5, DCLRE1B, ADNP, MCM4, DTL, MSH6, NRF1, HMGXB4, ZNF367, UNG, HNRNPM, SRSF1, TARDBP, RPA1, SENP1, USP1, RFWD3, RBMX, HNRNPU, PRIM1, UHRF1*

**Table 4:** GO enrichment analysis and the significant terms (biological processes)

Term	Count	*P-*value
GO:0006260 ~ DNA replication	21	5.08E-27
GO:0006270 ~ DNA replication initiation	10	8.09E-16
GO:0000082 ~ G1/S transition of mitotic cell cycle	12	3.50E-14
GO:0006281 ~ DNA repair	11	5.78E-09
GO:0006268 ~ DNA unwinding involved in DNA replication	5	2.47E-08
GO:0042769 ~ DNA damage response, detection of DNA damage	5	6.48E-06
GO:0032508 ~ DNA duplex unwinding	5	1.46E-05
GO:0016447 ~ somatic recombination of immunoglobulin gene segments	3	3.39E-05
GO:1901796 ~ regulation of signal transduction by p53 class mediator	6	6.24E-05
GO:0006974 ~ cellular response to DNA damage stimulus	7	7.21E-05
GO:0000398 ~ mRNA splicing, via spliceosome	7	1.03E-04
GO:0000722 ~ telomere maintenance via recombination	4	1.72E-04
GO:0019985 ~ translesion synthesis	4	2.45E-04
GO:0036297 ~ interstrand cross-link repair	4	6.12E-04
GO:0007049 ~ cell cycle	6	8.35E-04
GO:0016446 ~ somatic hypermutation of immunoglobulin genes	3	0.001318
GO:0070987 ~ error-free translesion synthesis	3	0.001865
GO:0042276 ~ error-prone translesion synthesis	3	0.001865
GO:0000083 ~ regulation of transcription involved in G1/S transition of mitotic cell cycle	3	0.002736
GO:0006297 ~ nucleotide-excision repair, DNA gap filling	3	0.002978
GO:0006397 ~ mRNA processing	5	0.003176

**Table 5:** GO enrichment analysis and the significant terms (cellular components)

Term	Count	*P*-value
GO:0005654 ~ nucleoplasm	50	1.78E-31
GO:0005634 ~ nucleus	44	3.49E-12
GO:0042555 ~ MCM complex	6	3.42E-11
GO:0000784 ~ nuclear chromosome, telomeric region	10	2.87E-10
GO:0016607 ~ nuclear speck	6	4.43E-04

**Table 6:** GO enrichment analysis and the significant terms (molecular functions)

Term	Count	*P*-value
GO:0003677 ~ DNA binding	23	1.21E-08
GO:0003678 ~ DNA helicase activity	6	1.62E-08
GO:0005515 ~ protein binding	51	4.03E-08
GO:0003697 ~ single-stranded DNA binding	7	7.63E-07
GO:0003682 ~ chromatin binding	10	6.83E-06
GO:0005524 ~ ATP binding	17	2.92E-05
GO:0042393 ~ histone binding	6	6.13E-05
GO:0003684 ~ damaged DNA binding	5	6.42E-05
GO:0004003 ~ ATP-dependent DNA helicase activity	4	1.78E-04
GO:0000166 ~ nucleotide binding	8	1.81E-04
GO:0003688 ~ DNA replication origin binding	3	6.26E-04
GO:0019899 ~ enzyme binding	7	9.62E-04
GO:0044822 ~ poly(A) RNA binding	12	0.001415
GO:0003723 ~ RNA binding	8	0.002595

**Table 7:** Significant KEGG signaling pathways

Term	Count	*P*-value
hsa03030: DNA replication	10	9.33E-15
hsa04110: Cell cycle	9	2.78E-08
hsa03430: Mismatch repair	5	2.13E-06
hsa03040: Spliceosome	6	2.05E-04
hsa03460: Fanconi anemia pathway	4	0.001369866

### PPI network construction and screening of hub genes

**Fig. 12:**
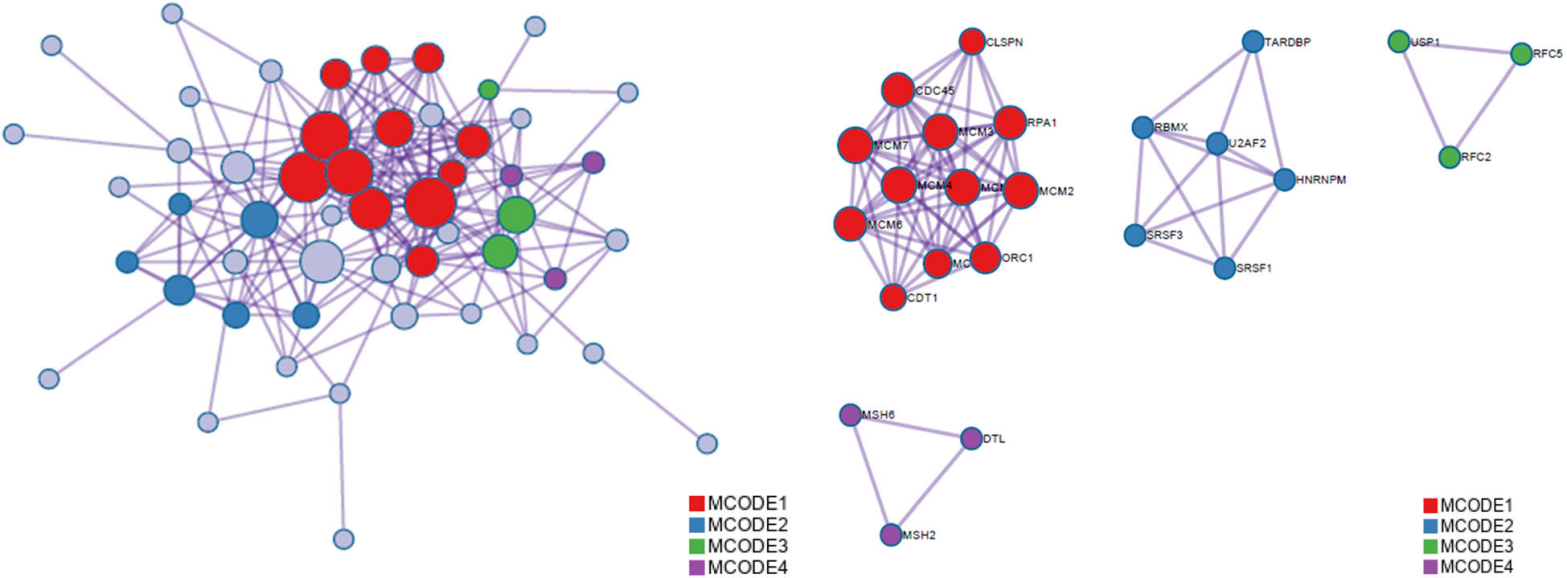
Protein-protein interaction network and MCODE components (Metascape)

**Fig. 13:**
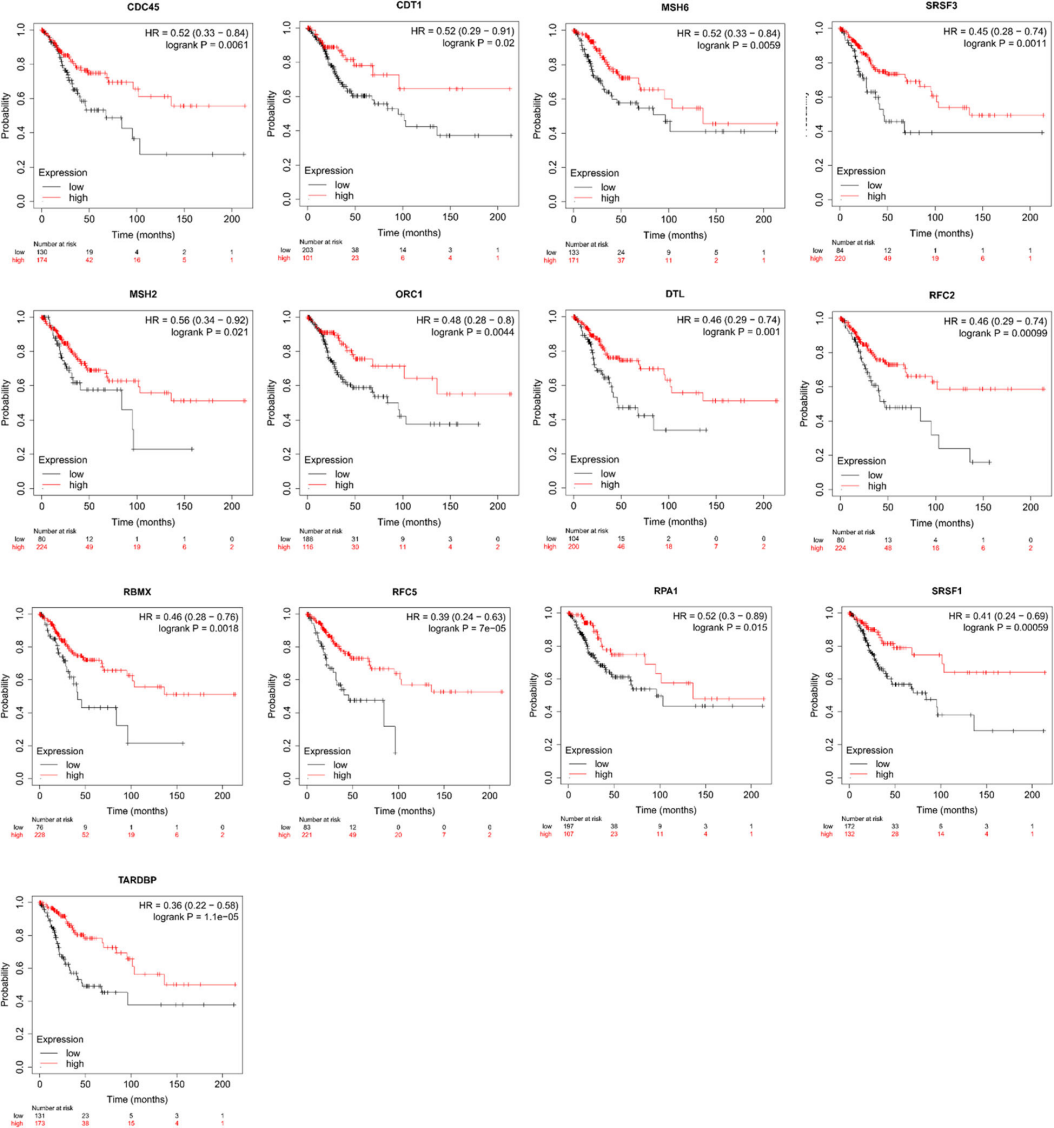
Prognostic value of the mRNA expression of the hub genes related to MCMs in CESC patients (Kaplan-Meier plotter database)

**Fig. 14:**
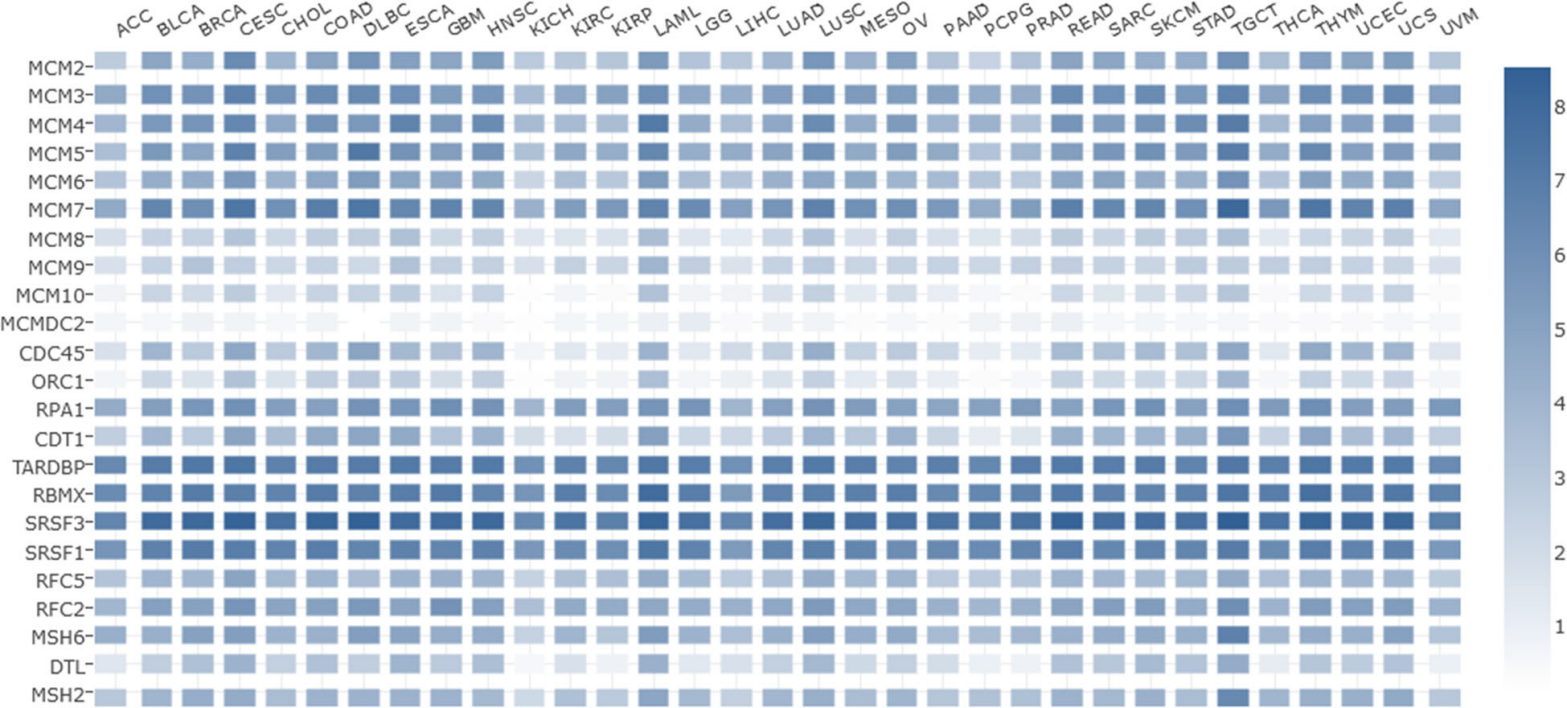
Interactive heat map analysis of tissue-wise expression in different cancer types (GEPIA2)

As shown in Fig. 12, the PPI network was successfully constructed. There were 4 Molecular Complex Detection (MCODE) components identified from the PPI network. MCODE 1 (*MCM2/3/4/5/6/7/8*, *CLSPN*, *CDC45*, *ORC1*, *RPA1*, and *CDT1*) played an important role in the activation of the prereplicative complex (R-HSA-68962), the activation of ATR in response to replication stress (R-HSA-176187) and DNA replication preinitiation (R-HSA-69002). MCODE 2 (*TARDBP*, *RBMX*, *U2AF2*, *HNRNPM*, *SRSF3*, and *SRSF1*) was important in the regulation of mRNA metabolic processes (GO:1903311) and spliceosomes (ko03040, hsa03040). MCODE 3 (*USP1*, *RFC5*, and *RFC2*) was important in the recognition of DNA damage by the PCNA-containing replication complex (R-HSA-110314), DNA damage response (GO:0042769) and PID Fanconi pathway (M1). MCODE 4 (*MSH6*, *DTL*, and *MSH2*) was important in the response to UV (GO:0009411), the response to light stimulus (GO:0009416) and DNA repair (R-HSA-73894). The survival analysis results of the hub genes from the 4 MCODE components are shown in Fig. 13 (the prognostic value of MCMs is shown in Fig. 8 and Fig. 9). The expression of *HNRNPM*, *U2AF2*, *USP1* and *CLSPN* showed no correlation with the prognosis of CESC patients, while the high expression of the other 13 genes was significantly related to a better prognosis. Moreover, higher mRNA expression of *RFC5*, *RFC2*, *DTL*, *RBMX*, *ORC1* and *MSH2* was significantly associated with longer OS in CESC patients. These results indicated that the hub genes might play an important role in cervical cancer and provide potential molecular targets. In addition, GEPIA2 was used to generate an interactive heat map of the expression of MCMs and related genes in different cancer types (Fig. 14). The hub genes and MCM2/3/4/5/6/7/8 might have potential clinical value for the survival and prognosis of cervical cancer patients.

## Discussion

Cervical cancer is the most common cancer among women worldwide. High-risk human papillomavirus (HPV) infection causes high morbidity and mortality. Therefore, the development of cervical cancer vaccines and screening technology and the exploration of clinical targets with good application prospects are still important. MCMs are implicated in the development of multiple cancers, including cervical cancer. Thus, MCM proteins have emerged as exceptionally promising markers for cervical cancer screening and early diagnosis [34]. Mitali Das et al. [35] explored the role of MCM4/5/6/10 in cervical cancer and their correlation with the clinical parameters of cervical cancer, and further study indicated that cervical cancer cells may use excess MCMs as a backup for replicative stress [36]. V N Saritha et al. [37] showed that MCM2/5 expression was upregulated in low-grade lesions, high-grade lesions and malignancies to a greater extent than p16 and p63. Gurjeet Kaur et al. [38] evaluated MCM gene expression profiles and MCM2 protein in HPV-associated cervical carcinogenesis. There is growing evidence that MCMs may be used as biomarkers to predict the malignant potential of cervical lesions. However, as cervical cancer is a complex disease involving many molecular interactions and complex signaling pathways, reports of MCMs and their related genes in cervical cancer are few and insufficient, and more research is still needed for the prevention and treatment of cervical cancer. In this study, bioinformatics was used to mine expression data and perform subsequent comprehensive analyses, which were based on a large amount of public data of patients with cervical cancer, for the in-depth study of the related molecular mechanisms of MCMs and protein molecular interactions, the prediction of related biomarkers, and the exploration of factors related to the good survival and prognosis of patients.

The expression of different MCMs in cervical cancer patients obtained from professional databases (ONCOMINE, UALCAN and HPA) showed that the mRNA and protein expression of MCMs increased in tumor tissue. These findings promoted our understanding of the expression of different MCMs in cancer patients. In particular, to evaluate and predict the potential clinical value of MCMs, analyses of individual cancer stages, the survival of cancer patients and the genetic alterations of different MCMs were performed using the UALCAN, Kaplan-Meier plotter and cBioPortal databases. The multidatabase analysis revealed that MCMs had great potential clinical significance. MCM2/3/4/5/6/7/8 might be used as potential indicators for survival in patients with cervical cancer, which needs more research and verification. Moreover, a high mutation rate (71%) of MCMs was observed in cervical cancer patients. *MCM2*, *MCM4*, *MCM8*, *MCM3* and *MCM7* ranked as the top five genes with the highest number of genetic alterations, but genetic alterations in MCMs were not significantly associated with longer OS or DFS in CESC patients. According to these results, the intervention strategy of mutating MCM genes to achieve longer survival times in patients might not be effective. However, controlling the gene transcription and protein expression of MCMs might be an effective intervention method for CESC patients.

After the expression analysis and clinical value evaluation of MCMs, the coexpressed genes and related genes of MCMs were screened by cBioPortal and GEPIA2 analysis, and then the systematic enrichment analysis of related genes was performed by Metascape and the DAVID database to deepen the understanding of the role of MCMs and related genes in cervical cancer. A total of 59 genes were involved in the enrichment analysis. The enrichment results revealed that these genes played an important role in DNA replication, the cell cycle, DNA repair, the DNA damage response, the regulation of signal transduction by p53 class mediators and other important biological processes. Moreover, these genes were significantly enriched in some important cellular components, such as the nucleoplasm, nucleus, MCM complex, nuclear chromosome, telomeric region and nuclear speck, which were also involved in DNA binding, DNA helicase activity, protein binding, ATP binding, nucleoside binding, DNA replication origin binding and RNA binding. We found that MCMs and their related genes were significantly enriched in some important pathways, such as the DNA replication (*RFC5*, *MCM7*, *RFC2*, *PRIM1*, *RPA1*, *MCM3*, *MCM4*, *MCM5*, *MCM6*, and *MCM2*), cell cycle (*RBL1*, *CDC45*, *MCM7*, *ORC1*, *MCM3*, *MCM4*, *MCM5*, *MCM6*, and *MCM2*), mismatch repair (*RFC5*, *MSH6*, *MSH2*, *RFC2*, and *RPA1*), spliceosome (*HNRNPM*, *U2AF2*, *SRSF1*, *HNRNPU*, *SRSF3*, and *RBMX*) and Fanconi anemia (*BRIP1*, *RPA1*, *USP1*, and *FANCC*) pathways. Further study of these pathways can deepen the understanding of the molecular mechanisms related to the occurrence and development of cervical cancer.

In the current study, genes coexpressed with MCMs and their related genes were successfully screened for PPI network construction. A total of 4 key subnetworks were screened. Survival analysis showed that 13 hub genes (*CDC45*, *ORC1*, *RPA1*, *CDT1*, *TARDBP*, *RBMX*, *SRSF3*, *SRSF1*, *RFC5*, *RFC2*, *MSH6*, *DTL*, and *MSH2*) from the key subnetworks might have potential clinical value. These genes and MCM2/3/4/5/6/7/8 were significantly related to the survival and prognosis of cervical cancer patients. Thus, our results might provide bioinformatics support for MCMs and their related genes in the prevention and clinical treatment of cervical cancer.

However, this study had three limitations. First, the data analyzed in this study were from public databases, and the analysis results might be affected by the quantity and quality of the data. Second, we did not evaluate the potential therapeutic or diagnostic effects of MCMs in detail. Finally, we did not explore the potential mechanisms of MCMs and hub genes in cervical cancer in detail, and the effect on prognosis requires follow-up data. Therefore, further research is needed to verify our findings and to explore the clinical application of MCMs and their related genes for the treatment of cervical cancer.

## Conclusion

In conclusion, this study used a variety of bioinformatics methods to explore the transcriptional expression of MCMs as potential indicators of survival in patients with cervical cancer, obtained target genes with potential application value, and deepened the understanding of the influence of MCMs and their related genes in cervical cancer. These genes can be used to diagnose the progression of the disease before it leads to cancer. Moreover, our findings promoted the understanding of the MCM protein family and clinically related molecular targets for cervical epithelial neoplasia and cervical cancer, which provided new insight into the biological functions of MCMs in cervical cancer.

## Data Availability

The data used for analysis were all from online public databases and could be retrieved. Based on these data, we conducted further analysis and research. The datasets generated and analyzed during the current study are available in the ONCOMINE database, UALCAN database, Human Protein Atlas database, Kaplan-Meier plotter database, and GEPIA2 database repository. Preliminary enrichment analysis, molecular interaction network construction and hub gene screening were performed via the Metascape website, and the DAVID database was used for further verification. The websites of the databases are as follows: [https://www.oncmine.org], [http://ualcan.path.uab.edu/], [https://www.proteinatlas.org/], [http://kmplot.com/analysis], [https://metascape.org/gp/index.html], [https://david.ncifcrf.gov/], and [http://gepia2.cancer-pku.cn].

## Abbreviations

MCM: minichromosome maintenance
MCMDC2: MCM domain-containing 2
GO: Gene Ontology
KEGG: Kyoto Encyclopedia of Genes and Genomes
PPI: protein-protein interaction
CESC: cervical squamous cell carcinoma
HPA: Human Protein Atlas
DAVID: Database for Annotation, Visualization and Integrated Discovery
TCGA: The Cancer Genome Atlas
OS: overall survival
DFS: disease-free survival
MCODE: Molecular Complex Detection
HPV: Human papillomavirus
CESCE: cervical squamous cell carcinoma epithelia
HG-CIN: high-grade cervical squamous intraepithelial neoplasia epithelia
